# Clinical anatomy of the musculoskeletal system in the hip region

**DOI:** 10.1007/s12565-021-00638-3

**Published:** 2021-10-22

**Authors:** Masahiro Tsutsumi, Akimoto Nimura, Keiichi Akita

**Affiliations:** 1 Department of Clinical Anatomy, Graduate School of Medical and Dental Sciences, Tokyo Medical and Dental University, 1-5-45 Yushima, Bunkyo-ku, Tokyo, 113-8519 Japan; 2 Inclusive Medical Science Research Institute, Morinomiya University of Medical Sciences, Osaka, Japan; 3 Department of Functional Joint Anatomy, Graduate School of Medical and Dental Sciences, Tokyo Medical and Dental University, Tokyo, Japan

**Keywords:** Clinical anatomy, Gluteus medius, Hip joint capsule, Hip stability, Iliofemoral ligament

## Abstract

Although the hip joint is regarded as inherently stable, hip pain and injuries caused by traumatic/non-traumatic hip instability are relatively common in active individuals. A comprehensive understanding of hip anatomy may provide better insight into the relationships between hip stability and clinical problems. In this review, we present our recent findings on the hip morphological characteristics, especially focusing on the intramuscular tendon of the gluteus medius tendon and its insertion sites, hip capsular attachment on the anterosuperior region of the acetabular margin, and composition of the iliofemoral ligament. We further discussed the hip stabilization mechanism based on these findings. The characteristics of the gluteus medius tendon suggest that even a single muscle has multiple functional subunits within the muscle. In addition, the characteristics of the hip capsular attachment suggest that the width of the capsular attachment is wider than previously reported, and its wide area shows adaptive morphology to mechanical stress, such as bony impression and distribution of the fibrocartilage. The composition of the iliofemoral ligament and its relation to periarticular structures suggest that some ligaments should be defined based on the pericapsular structures, such as the joint capsule, tendon, and aponeurosis, and also have the ability to dynamically coordinate joint stability. These anatomical perspectives provide a better understanding of the hip stabilization mechanism, and a biomechanical study or an in vivo imaging study, considering these perspectives, is expected in the future.

## Introduction

Although the hip joint is one of the most inherently stable joints (Neumann 2020), hip pain and injuries, caused by hip instability are problematic and common in active individuals (Kalisvaart and Safran 2015; Kemp et al. 2020; Mosler et al. 2015; Safran 2019). The management of hip pain and injuries has recently evolved with remarkable advances in imaging modalities and arthroscopic techniques (Glick et al. 2014; Lynch et al. 2013). Since clinicians have to fully interpret the information gained from new technologies, a comprehensive understanding of the hip anatomy may help its interpretation and promote further evolution in hip management.

We investigated the musculoskeletal system in the hip region while focusing on the relationships between the intramuscular tendon and its bony attachment morphology, characteristics of the capsular attachment, and composition of the ligament. In this review, we present our research on the musculoskeletal system in the hip region and discuss insights into the hip stabilization mechanism based on the findings.

## Functional subunit within the gluteus medius

Clinical problems, such as a tear of the gluteus medius tendon, caused by its morphological characteristics, are a good example for reconsidering the hip stabilization mechanism of the muscle. The gluteus medius is the largest muscle among the hip abductor muscles and is vital for hip stability (Neumann 2010). Recently, tears of the gluteus medius tendon have been recognized as a potential cause of lateral hip pain, which is a relatively common clinical symptom (Pierce et al. 2018; Segal et al. 2007; Zhu et al. 2020). According to previous reports, tears of the anterior fibers of the gluteus medius tendon occur more frequently than those of the posterior fibers (Bunker et al. 1997; Connell et al. 2003; Davies et al. 2013). The relationships between the different frequencies of the tendinous tears within the muscle and the morphology of the gluteus medius may be important to facilitate early diagnosis with imaging, appropriate surgery, or rehabilitation protocols; however, few studies have focused on these relationships.

As originally described as “Wolff’s law” (Wolff 1892), the bony morphology is adaptive to the muscular power loaded on it via the tendon and, some previous studies have also shown the example of the bony anatomical structure (Nozaki et al. 2015; Sato et al. 2018; Tamaki et al. 2014). Therefore, the bony morphology of the gluteus medius may be important in considering the relationship between the frequency of tears and morphology of the gluteus medius tendon. Moreover, the intramuscular tendon may also be vital to its relationships because intramuscular tendons with different characteristics, such as the length and thickness, might form a single myotendinous unit and its characteristics could reveal the accurate tendinous compositions and detail footprints (Arai et al. 2008; Mochizuki et al. 2008; Nimura et al. 2014; Sato et al. 2012, 2018).

**Fig. 1:**
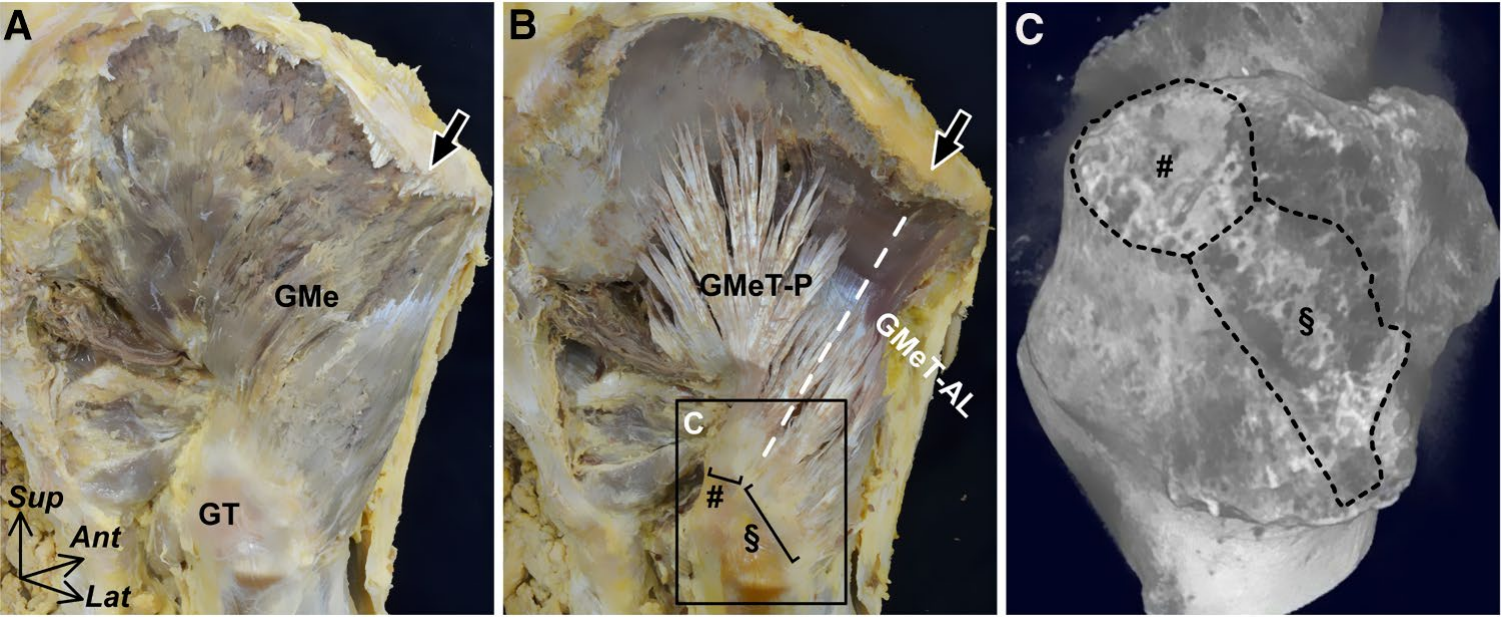
Intramuscular tendon of the gluteus medius and its insertion sites. Posterolateral aspects of the right hip. **A** Gluteus medius (GMe) mainly originates from the outer surface of the ilium, and the arrow indicates the inflection point on the iliac crest between the posterior and anterolateral parts of the outer surface of the ilium. **B** After removal of the muscular portion of the GMe, the posterior and anterolateral parts of the intramuscular tendons (GMeT-P and GMeT-AL, respectively) can be identified based on this inflection point (arrow). The number and section signs indicate the insertion of the GMeT-P and GMeT-AL on the greater trochanter (GT), respectively. **C** Micro-CT image of the greater trochanter (GT) of the boxed region in B. Insertion of GMeT-P (number sign) and GMeT-AL (section sign) can be identified as the superolateral and lateral facets of the GT, respectively. *Ant* anterior, *Lat* lateral and *Sup* superior. ( Modified from: Tsutsumi et al. 2019a)

Regarding the bony morphology, the gluteus medius mainly originates from the outer surface of the ilium, which is composed of two parts, the posterior and anterolateral parts, based on the directions of the surface of the ilium (Fig. 1A). Based on this bony morphology, the posterior and anterolateral parts of the intramuscular tendons can be roughly distinguished (Fig. 1B). The posterior part is long and thick and converges on the superoposterior facet of the greater trochanter, which can be identified using microcomputed tomography (micro-CT) (Fig. 1C). The anterolateral part is short and thin and runs posteroinferiorly toward the lateral facet of the greater trochanter, also identifiable by micro-CT. Therefore, the gluteus medius is regarded as having two subunits within the muscle (posterior and anterolateral parts) based on the bony morphology and intramuscular tendons (Tsutsumi et al. 2019a).

Concerning the frequency of tendinous tears within the muscle, the thin anterolateral part may be more prone to tears than the thick posterior part. This morphological suggestion corresponds to the clinical frequency of tears (Bunker et al. 1997; Connell et al. 2003; Davies et al. 2013). In addition, the convergence and thickness of the posterior part may be vital in hip stability, as indicated by electromyographic studies (Gottschalk et al. 1989; Semciw et al. 2013). Based on the corresponding relationships between the morphology and function of the subunits within the gluteus medius, the perspective that even a single muscle has multiple functional subunits within the muscle may provide better insights into the hip stabilization mechanism.

## Capsular attachment on the anterosuperior acetabular margin

Capsular attachment of the hip joint on the acetabular margin is illustrated as linear in an anatomical textbook (Gray 1985). On the other hand, the iliofemoral ligament is regarded as the major stabilizer of the hip joint, and it proximally and widely attaches to the inferior area of the anterior inferior iliac spine (AIIS), which is located in the anterosuperior region of the acetabular margin (Neumann 2020). Regardless of the proximity of the acetabular margin, the relationships between the anterosuperior capsule and iliofemoral ligament are often ambiguously described. For example, the iliofemoral ligament is described as “an accessory band of” or being “formed on,” “spreads triangularly on,” or “reinforced” the anterosuperior capsule (Braus and Elze 1954; Gray 1858; Neumann 2020; Schafer and Thane 1894). Ambiguity regarding its relationships may be related to a lack of precise knowledge of capsular attachment in the anterosuperior region of the acetabular margin.

In other articular systems, the joint capsule is thought to have a thin structure. For example, in the shoulder joint, Clark and Harryman (1992) stated that the joint capsule, which is located in the deepest layer of the rotator cuff, is a thin continuous sheet of interwoven collagen fibrils, and its attachment to the humerus was also illustrated as linear in an anatomical textbook (Gray 1985). However, Nimura et al. (2012) concluded that the conventional anatomical study overestimated the attachment width of the rotator cuff, and the capsular attachment of the shoulder joint occupied a substantial area of the greater tuberosity by the precise discrimination between the actual cuff insertion and capsular attachment. In other articular systems apart from the shoulder joint, such as the elbow, wrist, thumb metacarpophalangeal joint, knee, and ankle, many anatomical studies have also shown that the capsular attachment is not always linear, and its width varies according to the bony location (Amaha et al. 2019; Nasu et al. 2018; Nimura et al. 2014; Saka et al. 2021; Sato et al. 2018; Shimura et al. 2016). Therefore, these anatomical studies suggest that the width of the capsular attachment is wider than previously reported in most articular systems.

**Fig. 2:**
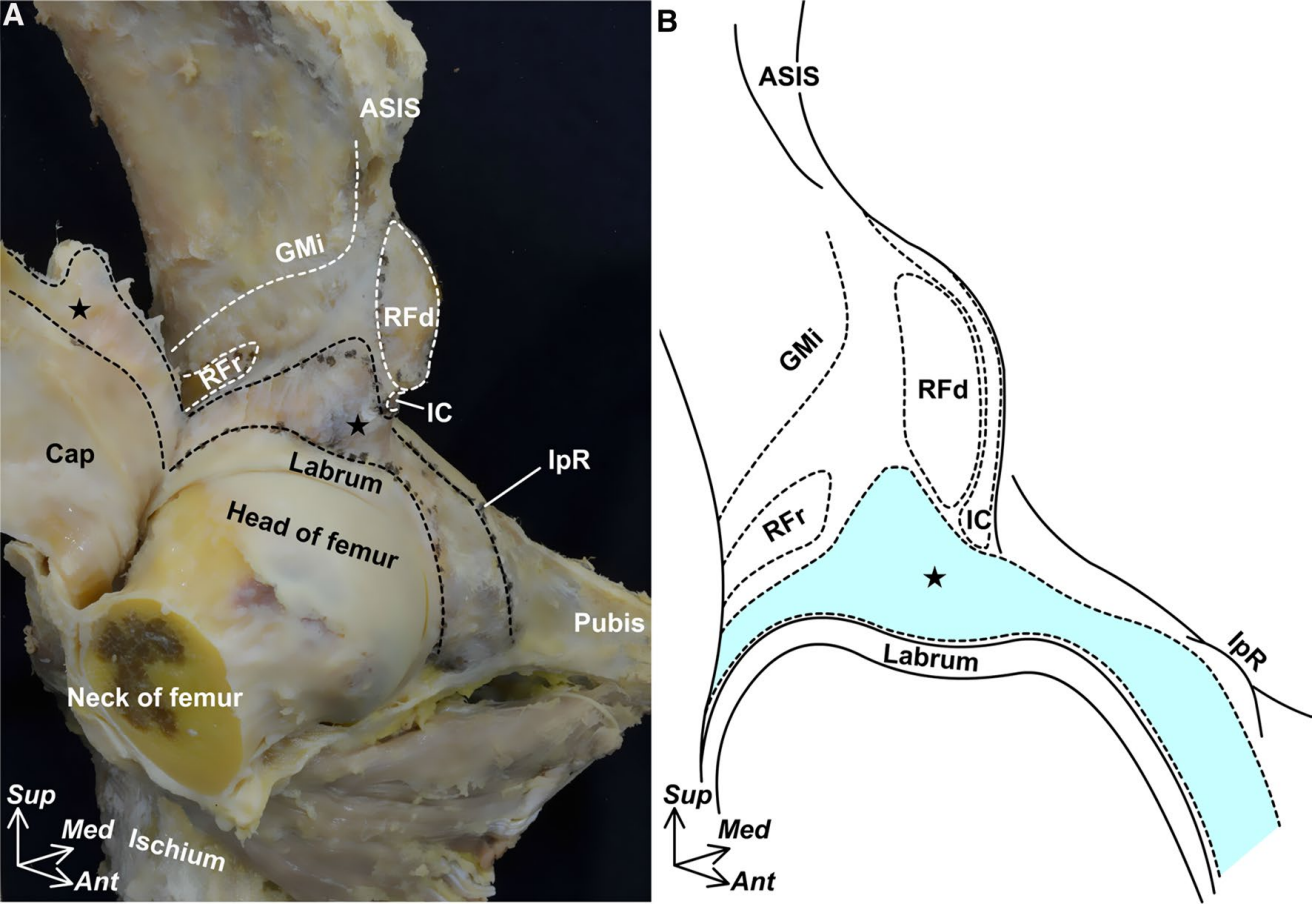
Attachment width of hip joint capsule on the anterosuperior region of the acetabular margin. Anterolateral aspects of the right hip. **A** The joint capsule (Cap) is detached from the acetabular margin, and the black dashed lines indicate its osseous attachment. The white dashed lines indicate the origin sites of the gluteus minimus (GMi), iliocapsularis (Ic), and the direct and reflected head of the rectus femoris (RFd and RFr, respectively). The star indicates the inferior area of the anterior inferior iliac spine. **B** Schematic illustration of the capsular attachment. *ASIS* anterior superior iliac spine, *IpR* iliopubic ramus, *Labrum* acetabular labrum, *Ant* anterior, *Med* medial, *Sup* superior. ( Modified from: Tsutsumi et al. 2019b)

**Fig. 3:**
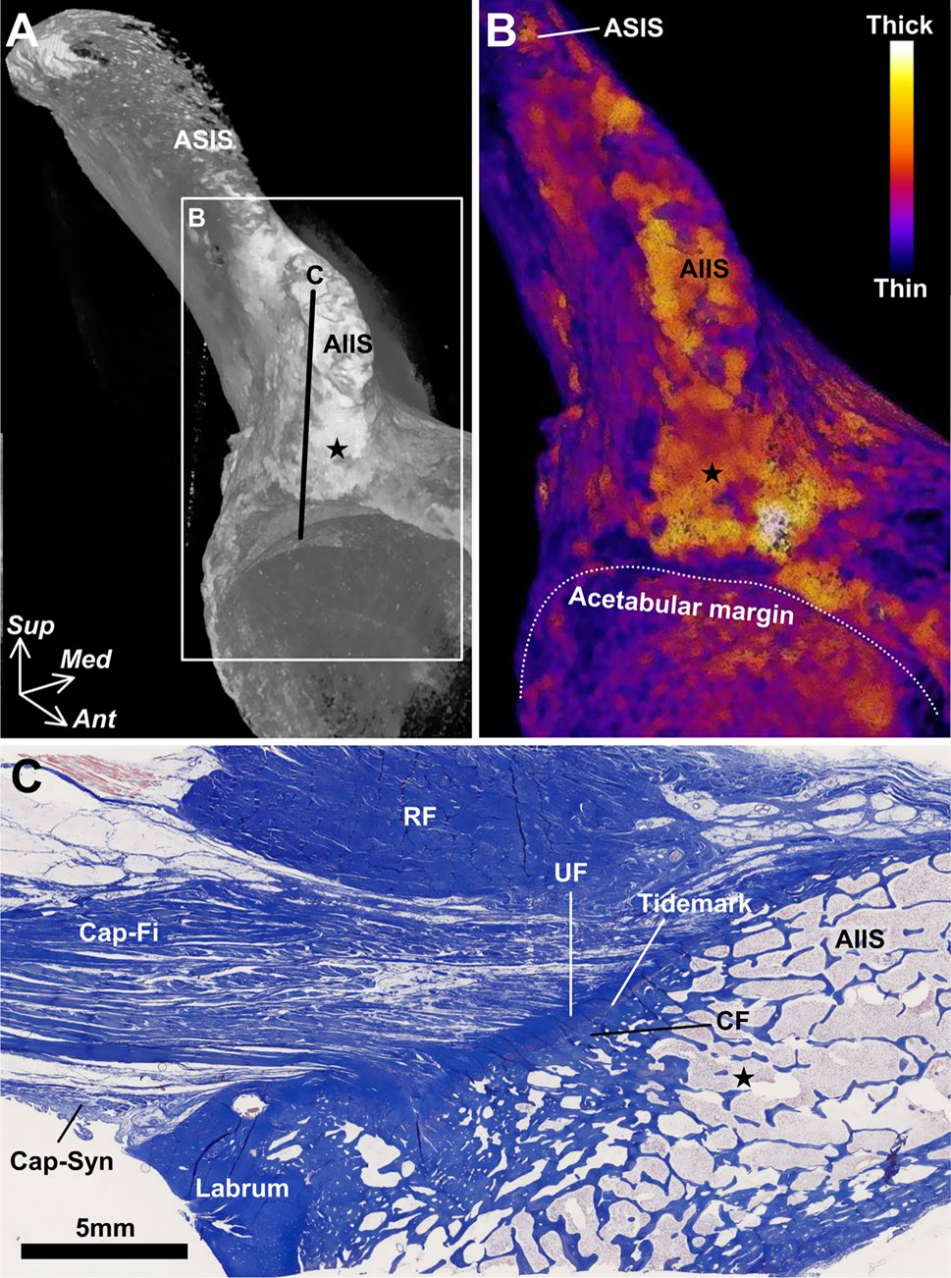
Bone morphology and histological characteristics of the capsular attachment inferior to the anterior inferior iliac spine. **A** Micro-computed tomography image of the anterolateral aspect of the right hip. The inferior area (star) of the anterior inferior iliac spine (AIIS) has a bony impression. **B** Cortical thickening maps of the boxed region in A is visualized after image processing by Bone J, which is ImageJ plug-in and can analyze the thickness of the structure of interest at a point (Hildebrand and Rüegsegger 1997; Doube et al. 2010). The brighter colors represent thicker cortical bone points. **C** Histological cross-section along line C in A is stained with Masson trichrome. The fibrous layer of the joint capsule (Cap-Fi) attaches to the inferior area of the AIIS via the fibrocartilage. *ASIS* anterior superior iliac spine, *Cap-Syn* synovial layer of the joint capsule, *CF* calcified fibrocartilage, *Labrum* acetabular labrum, *RF* rectus femoris, *UF* uncalcified fibrocartilage, *Ant* anterior, *Med* medial, *Sup* superior. ( Modified from: Tsutsumi et al. 2019b)

Regarding the hip capsular attachment, the width also varies according to the location, and the attachment width on the inferior area of the AIIS is larger than that on the anterosuperior region of the acetabular margin (Fig. 2). Moreover, the capsular attachment on the inferior area of the AIIS corresponds to the bony impression, which is identified by micro-CT (Fig. 3A). This bony impression on the inferior area of the AIIS shows cortical bone thickening (Fig. 3B). These bony morphological features, particularly cortical bone thickening, are reported to correspond to the high tensile stress from dense connective tissues (Horiuchi et al. 2020; Tano et al. 2021). In addition, histological analysis revealed that the ligament-like structure independent of the joint capsule did not exist in the inferior area of the AIIS, and the joint capsule attaches to the inferior area of the AIIS via the fibrocartilage (Fig. 3C). As previously described, the distribution of fibrocartilage at the attachment site correlates highly with the levels of mechanical stress (Benjamin and Ralphs 1998). Therefore, the capsular attachment on the inferior area of the AIIS is highly adaptive to mechanical stress based on its attachment width, bony morphology, and histological features, and can be regarded as identical to the origin of the iliofemoral ligament (Tsutsumi et al. 2019b).

This anatomical knowledge of the hip capsular attachment highlights important clinical insights. Although hip instability after hip arthroscopy is generally considered a rare complication (Harris et al. 2013; Kowalczuk et al. 2013), its prevalence has increased in recent years (Ramos et al. 2017; Yeung et al. 2016). During some surgical procedures of hip arthroscopy, the joint capsule is partially detached from the inferior area of the AIIS for labral repair (Fry and Domb 2010; Krych et al. 2013) and AIIS decompression (Hapa et al. 2013; Michal et al. 2020), probably because its attachment is thought to be a linear structure. However, as mentioned above, the capsular attachment inferior to the AIIS, which is identical to the origin of the iliofemoral ligament, is highly adaptive to mechanical stress. Therefore, the detachment of the capsular attachment inferior to the AIIS during hip arthroscopy might have a great impact on hip instability, as also indicated by a biomechanical study (Fagotti et al. 2020), and may be related to an increase in the prevalence of hip instability after hip arthroscopy in recent years. Based on the morphological features in the area with wide capsular attachment, such as the bony impression and distribution of the fibrocartilage, as well as the hypothetical relationships between capsular detachment and hip instability, the perspective that the width of the capsular attachment is wider than previously reported is vital in considering joint stability.

## Dynamic stabilization mechanism via the iliofemoral ligament

As described above, the capsular attachment on the inferior area of the AIIS is highly adaptive to mechanical stress and identical to the origin of the iliofemoral ligament (Tsutsumi et al. 2019b). Generally, the iliofemoral ligament spreads distally on the anterosuperior region of the hip joint, in an inverted Y, and is composed of the transverse and descending parts (Neumann 2020; Schafer and Thane 1894). The transverse part extends to the tubercle of the femur at the superolateral end of the intertrochanteric line and descending part to the inferomedial end of the line (Neumann 2020; Schafer and Thane 1894). Although the bony attachment of the iliofemoral ligament has been well investigated (Tamaki et al. 2020; Telleria et al. 2014; Wagner et al. 2012), the positional relationships between the iliofemoral ligament and the periarticular structures, such as the joint capsule or the hip muscles, have not been fully investigated.

Although the ligament is often assumed to be a bundle-like structure connecting bone to bone, drawing an arbitrary border between the ligament and periarticular structures is not appropriate in some cases. According to some histological textbooks, the ligament is classified as dense connective tissue, and its fibers are less regularly arranged than those of tendons, which are oriented parallel to the long axis, and more regularly than those of aponeuroses (Fawcett and Bloom 1986; Pawlina and Ross 2016). These fiber arrangements of the ligament are determined according to the local tensional demands, namely the relation to the surrounding anatomical structures, such as the joint capsule, tendon, or aponeurosis of the pericapsular muscles (Schleip 2012). For example, the humeroulnar joint capsule could not be separated from the tendinous septum between the flexor pronator muscles and these deep aponeuroses, and some of these complexes were the anterior bundle of the ulnar collateral ligament (Hoshika et al. 2019). In other articular systems apart from the elbow, such as knee and ankle, recent anatomical studies have also shown that the so-called “ligament” can be precisely defined based on pericapsular structures, such as the joint capsule, tendon, and aponeurosis (Amaha et al. 2019; Nasu et al. 2020).

**Fig. 4:**
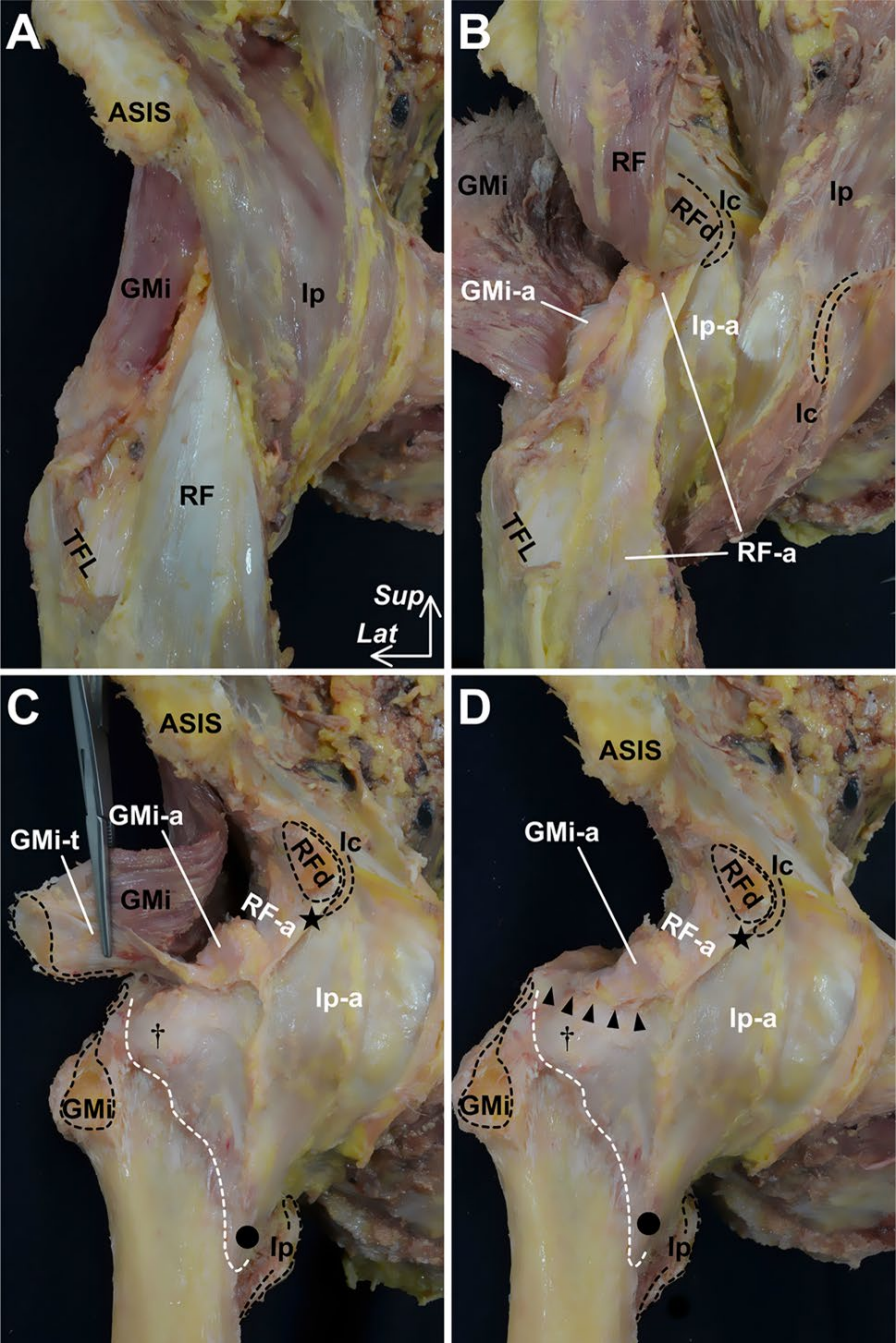
Spatial geometry of the gluteus minimus, iliopsoas and hip joint capsule. Anterior aspects of the right hip. **A** Surface of the pericapsular muscles, including the gluteus minimus (GMi), iliopsoas (Ip), and rectus femoris (RF). **B** The muscles are reflected to identify their deep aponeuroses. **C** Some of the deep aponeuroses, which do not connect to the outer surface of the joint capsule, are removed. The deep aponeuroses of the GMi (GMi-a), Ip (Ip-a), and proximal aponeurosis of the rectus femoris (RF-a) are connected to the joint capsule. In addition, both the rectus femoris and iliopsoas are also removed because they do not attach to the joint capsule, except for the deep aponeuroses. After detaching the GMi insertion on the femur, the connection between the GMi tendon (GMi-t) and the joint capsule can be identified. **D** Arrowheads indicate the cut line of the connection between the GMi-t and the joint capsule. The lateral end of the connection is adjoined to the tubercle of the femur at the superolateral end of the intertrochanteric line (dagger). The inferomedial end of the anterior border of the Ip-a corresponds to the inferomedial end of the intertrochanteric line (circle). *ASIS* anterior superior iliac spine, *Ic* iliocapsularis, *RFd* direct head of the RF, *Star* inferior area of the anterior inferior iliac, *TFL* tensor fasciae latae; white dashed line, distal margin of the joint capsule on the intertrochanteric line, *Lat* lateral and *Sup* superior. ( Modified from Tsutsumi et al. 2020)

**Fig. 5:**
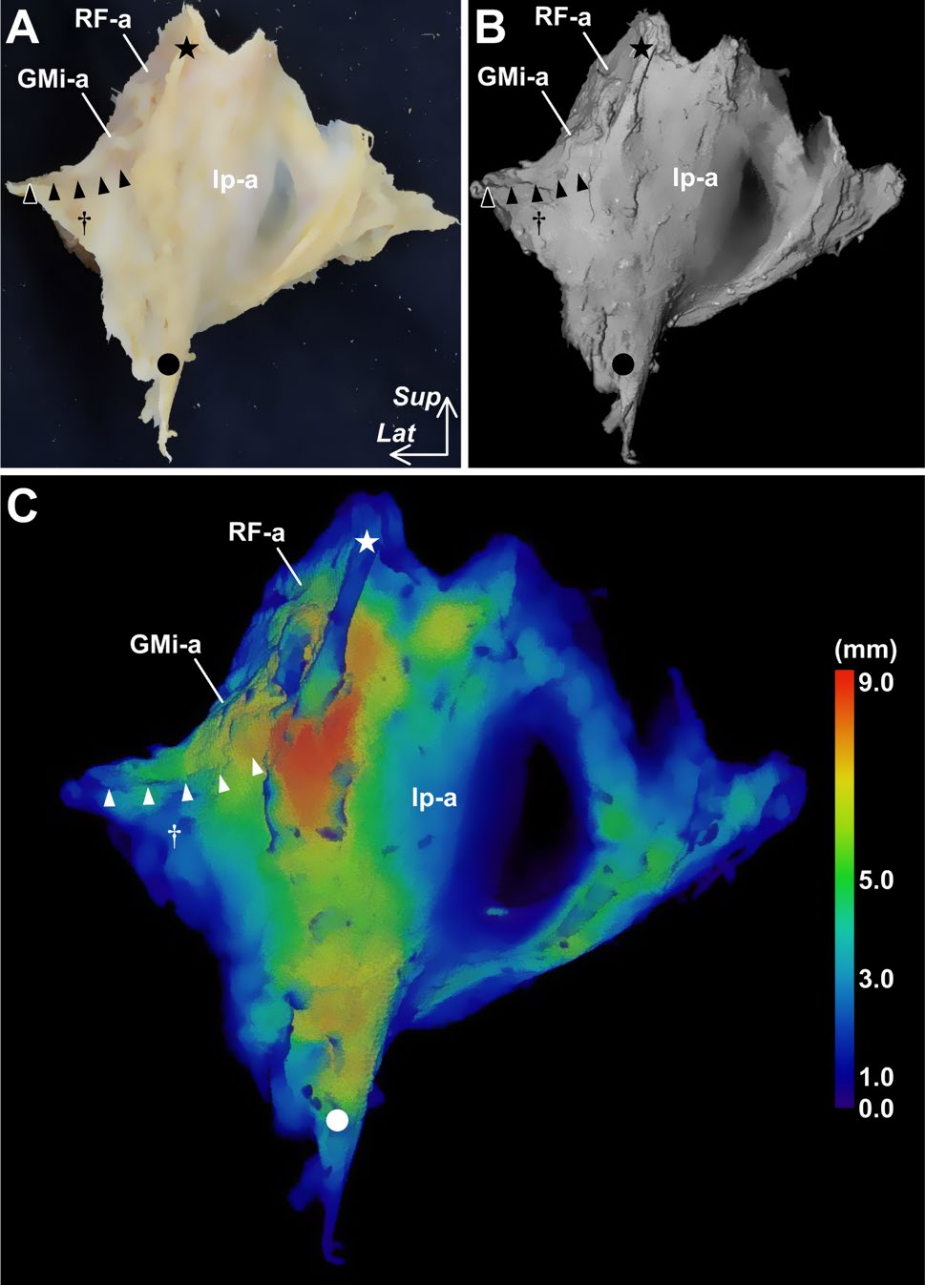
Outer appearance of the hip joint capsule and its thickness distribution. Anterior aspects of the hip joint capsule. **A** The hip joint capsule is detached from both the acetabular margin and femur while maintaining the three-dimensional (3D) morphology without cutting its substantial part. **B** 3D recontraction micro-CT image of A. **C** Thickness distribution map of B is visualized after image processing by Bone J, which analyzes the thickness of the structure of interest at a point including more than just bones (Hildebrand and Rüegsegger 1997; Doube et al. 2010). The color bar represents the approximate thickness corresponding to different colors. Arrowheads = cutting line of the connection between the gluteus minimus tendon and the joint capsule; circle = inferomedial end of the intertrochanteric line; dagger, superolateral end of the intertrochanteric line; *Ip-a* deep aponeurosis of the iliopsoas, *GMi-a* deep aponeuroses of the gluteus minimus, *RF-a* proximal deep aponeurosis of the rectus femoris, *Star* inferior area of the anterior inferior iliac spine, *Lat* lateral and *Sup* superior (Modified from Tsutsumi et al. 2021)

Concerning the anterosuperior region of the hip joint, on which the iliofemoral ligament is considered to be located, the gluteus minimus and iliopsoas are located immediately superficial to the joint capsule (Fig. 4A). The gluteus minimus tendon is connected to the joint capsule (Fig. 4B and C), and the lateral end of this connection is adjoined to the tubercle of the femur at the superolateral end of the intertrochanteric line (Fig. 4D). The deep aponeurosis of the iliopsoas is also connected to the joint capsule, and the inferomedial end of the anterior border corresponds to the inferomedial end of the intertrochanteric line. In addition, capsular thickening is identified in these connecting regions (Fig. 5). Therefore, the transverse and descending parts of the iliofemoral ligament could be regarded as joint capsules with fibers arranged according to their connection with the gluteus minimus tendon and deep aponeurosis of the iliopsoas, respectively (Tsutsumi et al. 2020).

This anatomical interpretation provides useful information regarding hip joint stability. In general, the iliofemoral ligament contributes to hip joint stability as the main static stabilizer (Myers et al. 2011; Walters et al. 2014). However, if the iliofemoral ligament is a joint capsule complex with a connection to the gluteus minimus tendon and deep aponeurosis of the iliopsoas, the iliofemoral ligament can dynamically coordinate the hip position during movement by transmitting the contraction force of the gluteus minimus and iliopsoas to the joint. Therefore, the iliofemoral ligament can be regarded as not only a static but also dynamic stabilizers. The precise definition of the ligament based on the pericapsular structures provides a new perspective that the so-called “ligament” has the ability to dynamically coordinate joint stability, which may provide better insights into the hip stabilization mechanism.

## Conclusion

This review highlights three anatomical perspectives. First, a single muscle has multiple functional subunits within the muscle. Second, the width of the capsular attachment is wider than previously reported. Finally, the so-called “ligament” has the ability to dynamically coordinate joint stability. These anatomical perspectives provide a better understanding of the hip stabilization mechanism, and a biomechanical study or an in vivo imaging study considering these perspectives is expected in the future.

## Data Availability

The datasets used and/or analyzed during the current study are available from the corresponding author on reasonable request.
